# Phytochemical Characterization and the First Report on the Antiproliferative Activity and Cytotoxicity of *Thymus fedtschenkoi* var. *handelii* (Ronniger) Jalas

**DOI:** 10.3390/ph19060844

**Published:** 2026-05-28

**Authors:** Tünay Karan, Ali Aydın, Çağrı Çağlar Sinmez, Ufuk Ülker, Ayşe Bulut, Mükerrem Betül Yerer, Bedrettin Selvi

**Affiliations:** 1 Department of Genetics, Faculty of Veterinary, Yozgat Bozok University, Yozgat 66200, Türkiye; 2 Department of Basic Medical Science, Faculty of Medicine, Yozgat Bozok University, Yozgat 66200, Türkiye; ali.aydin@yobu.edu.tr; 3 Department of History of Veterinary Medicine and Deontology, Faculty of Veterinary Medicine, Erciyes University, Kayseri 38039, Türkiye; cagrisinmez@erciyes.edu.tr; 4 Department of Microbiology, Faculty of Veterinary Medicine, Yozgat Bozok University, Yozgat 66200, Türkiye; ufuk.ulker@bozok.edu.tr; 5 Department of Physiology, Faculty of Dentistry, Yozgat Bozok University, Yozgat 66200, Türkiye; draysebulut@gmail.com; 6 Department of Pharmacology, Faculty of Pharmacy, Erciyes University, Kayseri 38039, Türkiye; mbyerer@erciyes.edu.tr; 7 Department of Biology, Faculty of Arts and Sciences, Tokat Gaziosmanpasa University, Tokat 60100, Türkiye; bedrettin.selvi@gop.edu.tr

**Keywords:** *Thymus fedtschenkoi* var. *handelii*, essential oils, GC-MS, MTT, cytotoxicity, good health and well-being

## Abstract

**Objectives**: This study aimed to investigate the phytochemical content of the endemic plant *Thymus fedtschenkoi* var. *handelii* (Ronniger) Jalas and, for the first time, to examine its anticancer potential on various cancer cell lines. **Methods**: The plant was collected from its natural habitat and the essential oils’ (EOs) composition was analyzed using GC-MS. The anticancer efficacy and cytotoxicity of plant extracts and the EOs were evaluated using 3-(4,5-dimethylthiazol-2-yl)-2,5-diphenyltetrazolium bromide (MTT) and lactate dehydrogenase (LDH) methods on lung (A549, Calu1, H1650), bone (SW1353, MG63, Saos2), prostate (PC3, DU145, LNCaP) and brain (A172, B35, C6) cancer cell lines, as well as normal cell lines (Beas2B, FL, HC). **Results**: The main components of the EOs were determined to be high amounts of carvacrol (51.12%), γ-gamma-terpinene (16.87%), and p-cymene (14.76%). Both the extract (GI_50_: 1.10–3.28 µg/mL) and the EOs (GI_50_: 1.05–2.03 µg/mL) exhibited strong antiproliferative activity. However, the EOs demonstrated markedly superior growth suppression, with TGI values of 1.97–9.19 µg/mL, whereas the extract required substantially higher concentrations (110.6–261.5 µg/mL). The LC_50_ values of all samples exceeded 500 µg/mL in all cell lines tested, indicating that the natural compounds predominantly had a cytostatic effect. Normal cells showed comparable reduced sensitivity, supporting selectivity. Morphological analyses further confirmed treatment-induced cellular alterations consistent with antiproliferative and apoptotic processes. Overall, essential oils emerged as the most effective fraction, combining low TGI values with moderate cytotoxicity and showing promise in in vitro activity. **Conclusions**: The potent and selective antiproliferative activity of *T*. *fedtschenkoi* var. *handelii* may hold significant therapeutic potential in the pharmaceutical industry.

## 1. Introduction

Herbal medicine continues to be widely used today, and its use is increasing every day. In particular, metabolites derived from plants possess effective pharmacological and biological activities. Essential oils (EOs) are secondary compounds, usually fragrant, obtained from the leaves, flowers, seeds, and roots of plants. EOs are oily substances that can be carried away by water vapor, have a strong scent, and give many plants their characteristic fragrance. They are called “volatile oils” because they can evaporate at room temperature when left exposed [1,2].

EOs are all hydrocarbons and are classified as phenylpropanoids, and as alcohol, ester, and aldehyde derivatives of terpenoids. The numerous compounds within them are separated and identified using a very powerful analytical technique called Gas Chromatography/Mass Spectrometry (GC/MS) [3]. EOs are primarily used in perfumery and cosmetics, and are also used as herbal teas and in the preparation of sauces for cooking. They are also used in animal feed as feed additives and to improve the digestibility of feeds. Due to the harm antibiotic-containing feed additives cause to animals and animal products, the use of antibiotics has been banned. Recently, there has been a growing trend towards using EOs with antibacterial properties as natural feed additives, replacing antibiotics [4,5]. EOs possess anticancer, anti-inflammatory, antioxidant, antifungal, antiseptic, antiparasitic, and insecticidal properties and are used for pharmaceutical purposes [6]. EOs obtained from thyme, rosemary, cinnamon, and clove plants have been reported to have antimicrobial, antiviral, antifungal, and antiparasitic properties due to their main components, such as carvacrol, thymol, cinnamaldehyde, ionone, and eugenol [7].

Cancer is a disease dependent on age, genetic factors, and environmental conditions, and is found in almost every part of the world and can be fatal. Treatment options include surgery, radiation, immunotherapy, chemotherapy, and targeted therapies [8]. Today, it has been scientifically established that natural compounds derived from plants play an important role in cancer treatment. EOs, in particular, may possess antiproliferative activity against cancer cells without exhibiting any toxic effects on healthy cells. Therefore, testing components derived from natural sources on cancer cells is very valuable [9,10].

*Thymus* sp. (thyme), belonging to the Lamiaceae family, is an important medicinal plant studied within the genus of aromatic plants. Compared to other plants, it has a richer content and quantity of essential oils [11]. The cytotoxicity of many thyme extracts and essential oils to various cancer cell lines has been determined. Ethanol extracts of *Thymus kotschyanus* were found to reduce viability in lung cell line A549 and cervical cancer cell line HeLa as the dose increased [12]. The cytotoxic effect of essential oil obtained from *Thymus vulgaris* L. against MDA-MB-231 cells has been investigated, and potential benefits against human triple-negative breast cancer have been identified [13]. Furthermore, another study found that *T. vulgaris* ethanol extracts had a significant cytotoxic effect on MCF-7 breast cancer cells [14]. Studies have shown that *T. fallax* EO, which has strong antioxidant activity, inhibits the growth of human DLD-1 colorectal cancer cells and has no cytotoxic effect on L929 fibroblast cells [15]. The EOs of *T. serpyllum* (IC_50_: 52.69 µg/mL), *T. algeriensis* (IC_50_: 62.53 µg/mL) have cytotoxic effects on MCF-7 cells [16]. Another study found that *T. convolutus* EO was effective against the colon cancer cell line (HT-29) [17].

Extensive research in the literature on *Thymus* sp. clearly demonstrates that EOs and extracts exhibit strong antioxidant, antimicrobial, and especially cytotoxic effects against various cancer strains [11]. However, phytochemical and biological activity studies on *Thymus fedtschenkoi* var. *handelii* (Ronniger) Jalas, a member of this genus with rich taxonomic diversity, are quite limited. Despite extensive research on the *Thymus* sp., *T. fedtschenkoi* var. *handelii* remains poorly characterized. The lack of data, particularly regarding the anticancer and cytotoxic potential of this variety, constitutes a significant gap in the literature. This research provides the first detailed evaluation of this variety’s therapeutic potential, contributing to the broader understanding of the *Thymus* sp. as a source of natural cytotoxic agents. Therefore, this study aimed to investigate the antiproliferative and cytotoxic activities of the EOs and extracts obtained from *T. fedtschenkoi* var. *handelii* in detail for the first time.

## 2. Results

### 2.1. EOs Content and Quantity

EOs of the *T. fedtschenkoi* var. *handelii* were analyzed using the GC-MS method. As shown in Table 1, the EOs were analyzed along with their broad retention times (RT). Mass spectra of the components were determined by comparing them to standards in the Adams and NIST library databases. Of the 36 components in the essential oils, carvacrol (51.12%), terpinene-gamma (16.87%), cymene (14.76%), carene (2.93%), and thymol (2.62%), were found to be the most abundant.

### 2.2. Antiproliferative Activity

Anticancer drug 5FU was used as a positive control and the TGI values of the plant extract in lung cancer cell lines (A549, Calu1, and H1650) were 255.9, 192.6, and 173.1 µg/mL, respectively (Table 2). The GI_50_ was 1.43, 2.09, and 2.34 µg/mL, and the LC_50_ was >500 µg/mL. The EO values for lung cancer cell lines were 23.08, 6.55, and 2.87 µg/mL for TGI, respectively (Table 2). GI_50_ was 2.03, 1.20, and 1.13 µg/mL, and LC_50_ was >500 µg/mL. Table 2 also presents the TGI, GI_50_, and LC_50_ values for the antiproliferative effects of the extract and EOs in bone cancer cell lines (SW1353, MG63, and Saos2), brain cancer cell lines (A172, B35, and C6), prostate cell lines (PC3, DU145, and LNCaP), and normal cell lines (Beas2B, FL, and HC). The EO group exhibited very low TGI values (1.97, 2.87 and 3.56 µg/mL, respectively), especially in Saos2 (Bone), H1650 (Lung) and PC3 (Prostate) cell lines (Table 2). The highest sensitivity was observed in Saos2 (bone cancer) cells with a GI_50_ value of 1.05 µg/mL. GI_50_ values of the extract group are also generally low (range 1.10–3.28 µg/mL) (Table 2).

The selectivity of the tested extract and EOs was further evaluated using the Tumor Selectivity Index (TSI), calculated as the ratio of the TGI value in normal cells to that in cancer cells. Although certain treatments exhibited potent antiproliferative activity, the TSI values indicated limited selectivity in several models. In particular, essential oils (EOs) demonstrated TSI values below or close to 1 in multiple cancer cell lines, including A549 (0.44), SW1353 (0.74), A172 (0.91), and LNCaP (1.10), suggesting comparable cytotoxic effects on both cancerous and normal cells. Moreover, the TGI value of EOs in Beas2B normal cells (1.16 µg/mL) was similar to or lower than that observed in several cancer cell lines. These findings indicate that, despite their strong antiproliferative activity, the tested EOs did not exhibit marked cancer selectivity under the present experimental conditions. Therefore, claims regarding selective anticancer activity and therapeutic applicability were moderated accordingly.

The IC_50_ concentrations of *Thymus fedtschenkoi* var. *handelii* extract and EOs were determined by testing increasing concentrations of each sample over a specific range on cells using the MTT method. The absorbance values obtained were then used to create a logarithmic curve, from which a logarithmic function was applied using Excel^®^ software (Supplementary Figure S1 and Table 3). The IC_50_ analysis revealed distinct differences in antiproliferative potency among the plant extract, EOs, and 5FU across all tested cell lines. The plant extract exhibited moderate activity, with IC_50_ values ranging from 63.8 to 82.8 µg/mL across cancer cell lines, indicating relatively consistent but limited potency (Table 3). In contrast, EOs demonstrated significantly enhanced activity, with IC_50_ values spanning a much lower range of 3.1 to 51.4 µg/mL, depending on the cell type (Table 3). The most pronounced effect was observed in the Saos2 osteosarcoma cell line, where EOs achieved an IC_50_ of 3.1 µg/mL, representing approximately a 25-fold increase in potency compared to the extract (77.5 µg/mL) (Table 3).

Across lung cancer models (A549, Calu1, H1650), EOs consistently outperformed both the extract and 5FU, with IC_50_ values of 51.4, 38.6, and 16.4 µg/mL, respectively, compared to extract values of 64.8–81.2 µg/mL and 5FU values of 72.4–83.1 µg/mL (Table 3). Similarly, in bone and cartilage cancer cell lines (SW1353, MG63, Saos2), EOs exhibited superior activity (IC_50_: 3.1–41.4 µg/mL) relative to extract (72.8–82.8 µg/mL) and 5FU (63.2–81.6 µg/mL). Brain cancer models (A172, B35, C6) also showed enhanced sensitivity to EOs (IC_50_: 24.6–35.8 µg/mL) compared to extract (63.8–72.8 µg/mL) (Table 3).

In prostate cancer cells (PC3, DU145, LNCaP), EOs maintained stronger activity (IC_50_: 23.6–35.9 µg/mL) than both extract (70.3–80.5 µg/mL) and 5FU (57.3–62.0 µg/mL) (Table 3). Notably, normal cell lines (Beas2B, FL, HC) exhibited IC_50_ values of 7.9–39.8 µg/mL for EOs, compared to 66.9–78.2 µg/mL for extract and 57.8–68.0 µg/mL for 5FU, indicating that EOs retain biological activity in non-cancerous cells, although sensitivity varied among cell types (Table 3). Overall, EOs consistently demonstrated 2–25 fold greater potency than the plant extract and, in many cases, outperformed 5FU, particularly in osteosarcoma and lung cancer models.

### 2.3. Cytotoxicity

Cytoplasmic lactate dehydrogenase activity was measured using an LDH cytotoxicity kit. The plant extract was found to have no cytotoxicity, showing efficacy below 10% in all cell lines. The cytotoxicity of the EOs was higher in all cell lines compared to the control drug 5FU. EOs have the highest cytotoxic activity in the table, with 18.8% in the Saos2 cell line. EOs also demonstrated high killing performance in LNCaP (prostate) (18.5%), DU145 (prostate) (18.2%), and SW1353 (bone) (18.0%) cells (Table 4).

### 2.4. Effect of EOs on Cell Morphology

**Figure 1:**
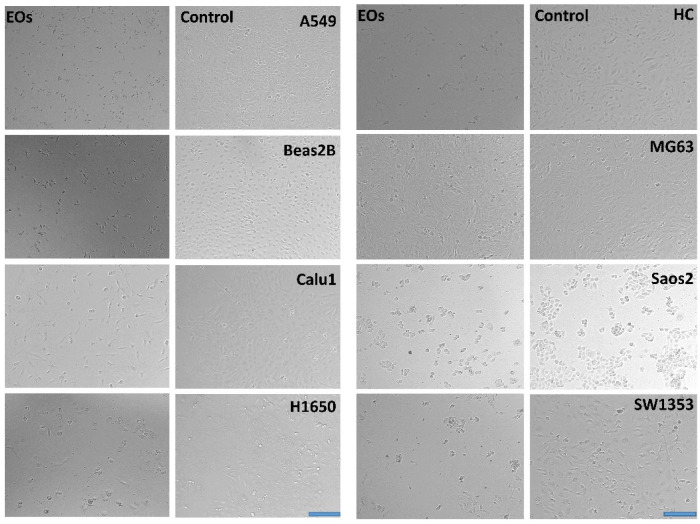
Effect of EOs on the morphology of A549, Beas2B, Calu1, H1650, HC, MG63, Saos2 and SW1353 cell lines. Bar is 100 µm.

**Figure 2:**
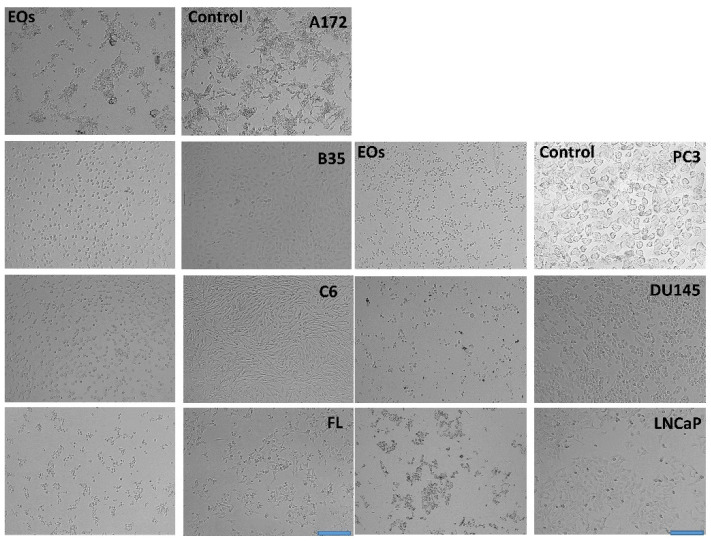
Effect of EOs on the morphology of A172, B35, C6, FL, PC3, DU145 and LNCaP cell lines. Bar is 100 µm.

The morphological effects of EOs with good anticancer activity on all tested cancer cell lines and normal lung cells (Beas2B), normal amnion cells (FL) and normal chondrocyte cells (HC) were investigated by inverse phase-contrast microscopy. The results were observed and photographed 24 h after application of the samples at the TGI concentration (Figure 1 and Figure 2). Morphological evaluation revealed distinct treatment-dependent cellular alterations. EO-treated cancer cells displayed prominent morphological hallmarks of apoptosis, including loss of spindle or epithelial morphology, transition to rounded, globular shapes, reduced cell density, cytoplasmic shrinkage, membrane blebbing, and detachment from the culture surface. In several cell lines (MG63, Saos2, SW1353), cells appeared fragmented, with apoptotic bodies and reduced intercellular connections. In contrast, control cells maintained typical morphology, characterized by adherent, elongated, or polygonal shapes with intact cell–cell contacts. Extract-treated cells showed milder morphological changes, primarily reduced confluence and slight rounding, consistent with their weaker antiproliferative effect. Normal cells also exhibited some degree of rounding and detachment upon EOs treatment, but their structural integrity was relatively better preserved than that of cancer cells.

## 3. Discussion

Herbal natural products, especially extracts, have been used in medicine since ancient times. Both the essential oils and the various extracts obtained from different *Thymus* species contain rich bioactive compounds (methanolic, ethanolic, aqueous, etc.), enabling them to exhibit selective cytotoxic effects on cancer cell lines. The most commonly studied EOs and extracts of *Thymus vulgaris* have been shown to be particularly effective against breast, colon, and lung cancer cells [18]. Methanolic extracts of *Thymus schimperi*, containing high amounts of the flavone luteolin and its derivatives, inhibited the growth of human gastric adenocarcinoma AGS cells with an IC_50_ of 88 μg/mL [19]. Cytotoxicity of *T. carnosus* hydroethanolic extract rich in salvianolic acid and rosmarinic acid was determined in MCF-7 cells (IC_50_: 86.87 µg/mL) [20].

Studies have shown that EOs obtained from different plants have anticancer effects on lung, liver, brain, prostate, breast, colon, and leukemia types of cancer. The EOs of Citrus aurantifolia have been found to have an antiproliferative effect by stimulating apoptotic mechanisms in human colon cancer cells (SW-480) [21]. *Cleistocalyx operculatus* EOs have shown antiproliferative effects in A549 (lung cancer), PC-3 (prostate cancer), MCF-7 (breast cancer), and A431 (skin cancer) cell lines [22].

*Thymus* sp. contain chemicals rich in EOs, flavonoids, and phenolics. The EOs of these species exhibit antimicrobial and antioxidant activities, particularly due to their high content of isomeric monoterpene phenols such as thymol and carvacrol. *Thymus capitatus* EOs was analyzed by GC-MS to contain thymol, p-cymene, carvacrol, and γ-terpinene at 62.3%, 10.9%, 6.7%, and 5.1%, respectively [23]. One study determined that *T. vulgaris*, *T. daenensis*, and *T. kotschyanus* were rich in thymol and carvacrol EOs, and that *T. kotschyanus* showed the highest cytotoxic activity against HeLa cells [24]. The EOs of *Thymus citriodorus*, whose main components are borneol (28.82%), thymol (14.43%), 3,7-dimethyl-1,6-octadiene-3-ol (8.26%), 1-methyl-4-[alpha-hydroxy-isopropyl]cyclohexene (8.23%) and the terpenes camphor (5.1%), strongly inhibited the liver cancer (HepG2) cell line with an IC_50_ of 0.34%. The anticancer potential of EOs depends not only on the quantity of molecules these contain, but also on their chemical structures and the synergistic interactions between these [25].

Although there have been some anticancer studies on *Thymus* sp., research on this valuable medicinal and aromatic plant is limited. No studies on the biological activity of the endemic *Thymus fedtschenkoi* have been found in the literature. In this study, the anticancer activity of the extract and essential oil of *T. fedtschenkoi* var. *handelii* was determined for the first time and compared with other species. In our study, carvacrol (51.12%), identified as the main component of *T. fedtschenkoi* var. *handelii* essential oil, differed from most species, such as *Thymus* sp. (*Thmus vulgaris*, *T. serpyllum*), which have thymol as their main component [26,27]. Of the 36 components representing 100% of *T. fedtschenkoi* var. *handeliide*, terpinene-gamma (16.87%) and cymene (14.76%) are the other main components. According to published data and our current study, chemical polymorphism exists among *Thymus* sp., and this variation results in different biological activities [28,29].

Our study evaluated the anticancer and cytotoxic effects of *T. fedtschenkoi* var. *handelii*. The TGI value refers to the concentration required to completely stop cell growth. The lower the value, the stronger the sample [30]. While the TGI values of the EOs group were between 1.97 and 9.19 µg/mL in most cancer lines, in the extract group, these values were much higher, between 110 and 255 µg/mL. This indicates that *T. fedtschenkoi* var. *handelii* EOs are much more effective than extracts in stopping cancer cell proliferation. EOs have even lower TGI values than the standard drug 5FU in many cell lines (Saos2 and PC3), indicating that their cytostatic (cell arresting) effect is quite high. The GI_50_ value indicates the concentration of a test substance required to inhibit cell growth by 50%. It is one of the most fundamental parameters used in cancer research to determine the potential of a substance as a drug candidate [31]. The GI_50_ values of the EOs group are very low, between 1.05 and 2.03 µg/mL, in almost all cancer cell lines. These values are quite similar to those of the standard chemotherapeutic drug 5FU (1.17–1.66 µg/mL), and in some lines (Saos2 and H1650), EOs have even been shown to inhibit growth by 50% at lower doses than 5FU. The most remarkable success was seen in the LNCaP (prostate cancer) cell line; here, the extract showed a more effective initial performance than 5-FU (1.25 µg/mL) at 1.10 µg/mL. LC_50_ represents the concentration required to kill 50% of cells (cytotoxic effect) [32]. The LC_50_ values for both extract and EOs were recorded as >500 µg/mL in most cell lines. While the cell growth-stopping (TGI) and -halving inhibition (GI_50_) values of the samples are very low, the fact that their direct cell-killing doses (LC_50_) are greater than 500 indicates that these substances have a wide safe working range.

In the MTT graph in Supplementary Figure S1, the substance concentration IC_50_ is seen at the point where cell viability drops from 100% to 50%. The lower this value, the more potent the plant extract or EO. The IC_50_ results further confirm the superior antiproliferative potency of EOs compared with the extract and 5FU. The EOs consistently exhibited lower IC_50_ values across most cancer cell lines, with particularly strong effects in Saos2 (3.1 µg/mL) and H1650 (16.4 µg/mL), indicating high sensitivity. In contrast, the extract showed weak activity (generally >60 µg/mL), whereas 5FU demonstrated moderate potency but was less effective than the EOs in most cases. However, the IC_50_ values in normal cells (Beas2B: 7.9 µg/mL) suggest limited selectivity. When interpreted together with TGI and LC_50_ data, EOs appear to exert strong cytostatic effects at low concentrations while inducing limited cell death (LC_50_ > 500 µg/mL). Consistent with these findings, morphological changes such as cell rounding, shrinkage, and membrane blebbing at IC_50_ levels support apoptosis-like mechanisms rather than necrosis. However, these observations alone are not sufficient to conclusively confirm apoptotic mechanisms. Since no molecular or biochemical apoptosis analyses, such as caspase activation, Annexin V staining, mitochondrial membrane potential assessment, or DNA fragmentation assays, were performed in the present study, the observed morphological changes should be interpreted only as indicative of possible apoptosis-related effects. Therefore, this limitation should be considered when interpreting the mechanistic implications of the findings, and further studies are required to validate the exact cell death pathways involved.

Table 3 shows the percentage of cytotoxicity of the substances that directly kill cells. 5FU generally exhibited a kill rate between 8.3% and 14.6%. EOs had the highest mortality rate across all cell lines (cancer and normal). Cell death rates generally ranged from 13.9% to 18.8%. The extract showed the lowest cytotoxicity values (1.2–9.8%). This confirms that the extract has a growth-slowing effect rather than killing cells. Low cytotoxicity is desired in normal cells (Beas2B, FL, HC). EOs also showed high cytotoxicity in normal cells (Beas2B: 17.3%, FL: 15.8%, HC: 16.9%). These values are very close to the killing rates of cancer cells, meaning they also affect healthy cells at a similar rate. The extract also exhibited low cytotoxicity in normal cells (4.1–8.8%), indicating a safer profile.

The existing literature indicates that carvacrol induces apoptosis (programmed cell death), particularly in prostate and bone cancer cells, which is consistent with our findings regarding PC3 (17.5%) and Saos2 (18.8%) cytotoxicity [33,34]. The high proportion of carvacrol in the EOs, when considered together with its biosynthetic precursors such as gamma-terpinene and p-cymene, suggests that the low IC_50_ values observed in Saos2 cell lines are due to the strong synergistic effect between these components. For all tested extracts and oils, the calculated LC_50_ values in normal cells (Beas2B, FL, HC) were above 500 µg/mL, proving that these natural components have low toxicity and can be used safely in therapeutic doses.

## 4. Materials and Methods

### 4.1. Plant and Extraction

*Thymus fedtschenkoi Ronniger* var. *handelii* (Ronniger) Jalas was collected in full bloom on June 28, 2023, from gypsum areas in Koçkaya village, Sason district of Batman, Turkey (38°24′06″ N, 41°17′23″ E), at an altitude of 1450 m. The species was identified by Dr. Bedrettin Selvi, and a voucher specimen with herbarium number (GOPU 9937) has been deposited in the Herbarium of the Faculty of Arts and Sciences at Tokat Gaziosmanpaşa University. Subsequently, 50 g of dried plant material was extracted in 300 mL of water at 50 °C for 2 h. After filtration, the water was removed under vacuum in a rotary evaporator, and crude extracts were obtained. The extraction yield was calculated as 45% (*w*/*w*).

### 4.2. Isolation of the Essential Oil

Briefly, 400 g of dried plant material was cut into small pieces, then placed in 2000 mL ground flasks, and 800 mL of pure water was added. EOs were obtained by hydrodistillation using a Clevenger apparatus (Calışkanlab, Ankara, Türkiye) by heating for 3 h. Until these were needed, the EOs were refrigerated.

### 4.3. GC-MS Analysis

Chemical analyses of the EOs were performed using Gas Chromatography/Mass Spectrometry (GC-MS) by Perkin Elmer Clarus 500 instrument ((PerkinElmer, Inc., Shelton, CT, USA) with a flame ionization detector (FID). Briefly, 20 mg of EOs was dissolved in 1.2 mL of acetone. A BPX5 column (30 m, 0.25 mm, 0.25 μm film thickness) was used. The injection volume was set to 2.0 μL and the injection temperature to 250 °C. Helium was used as the carrier gas with a flow rate of 1 mL/min and a dispersion ratio of 50:1. The oven program was started at 50 °C and heated to 100 °C at a rate of 5 °C per minute and held at this temperature for 2 min. Then, the temperature was increased by 3 °C per minute to 220 °C and held at this temperature for another 2 min. The total program time was set to 30 min, and the ionization energy was set to 70 eV. The elucidation of the compounds was determined by comparison with the library (NIST, Wiley, and Pfleger) based on retention time [35].

### 4.4. Cancer Lines and Cell Culture

A549 (ATCC, CCL-185), Calu1 (ATCC, HTB-54) and H1650 (ATCC, CRL-5883) lung cancer cell lines, SW1353 (ATCC, HTB-94), MG63 (ATCC, CRL-1427) and Saos2 (ATCC, HTB-85) bone cancer cell lines, PC3 (ATCC, CRL-1435), LNCaP (ATCC, CRL-1740) and DU145 (ATCC, HTB-81) prostate cancer cell lines, A172 (ATCC, CRL-1620), C6 (ATCC, CCL-107) and B35 (ATCC, CRL-2754) brain cancer cell lines, FL (ATCC^®^ CCL62) normal amnion, Beas2B (RRID, CVCL-0168) normal lung and HC (Sigma Aldrich, 402-05A, San Diego, CA, USA) normal chondrocyte cell line were used. All cell preparation procedures were performed in a sterile environment in a laminar flow cabinet. Cell lines were used after confluentization in DMEM or RPMI 1640 medium containing 10% FBS and 2% PenStrep solution at 37 °C, 5% CO_2_. Measurement plates were prepared with 10,000 cells per well. After approximately 16 h of pre-incubation, test molecules were added, and measurements were taken after 24 h of incubation [36].

### 4.5. MTT Assay

MTT [3-(4,5-dimethyl-thiazol-2-yl)-2,5-diphenyl tetrazolium bromide] test was used to measure the effects of EOs and extract on cell proliferation This test protocol was applied after incubating cancer cell lines with the test substances for 24 h. To completely dissolve the formazan crystals formed after incubation, 200 μL of DMSO was added to the medium and incubated for another 30 min. All samples were tested three times. Absorbance values were then determined by reading at 570 nm and 630 nm (reference) using an ELISA reader (Thermo Fisher, Waltham, MA, USA). 5FU was used as the standard.

### 4.6. GI50, TGI, LC50, and IC50 Parameter Calculations

Using XLfit5 software (version 5.3, IDBS, Guildford, UK), the extract and EOs half-maximal inhibitory concentration (IC50) and 5FU were determined as in Equation (1):

Inhibition (50%) = {(A − B)/B} × 100(1)

where A is the absorbance of the samples and B the solvent control.

The following formulas were used to measure NCI-60 survival parameters:

Growth Inhibition 50% (GI_50_) {(Ti − Tz)/(C − Tz)} × 100 = 50 (provided that Ti ≥ Tz) (2)

Total growth inhibition (TGI): Ti = Tz(3)

Lethal Concentration 50% (LC_50_) = {(Ti − Tz)/Tz} × 100 = −50 (provided that Ti < Tz) (4)

Here, Tz: zero point; C: control growth; Ti: inhibition of the test substance.

### 4.7. Cytotoxicity Test

The manufacturer’s instructions for the lactate dehydrogenase (LDH) assay were followed. Formazan production was used to measure LDH activity. LDH activity was determined as absorbance at 492 and 630 nm using a microplate reader (Thermo Fisher, Waltham, MA, USA). Cytotoxic activity was calculated using Equation (5) [37].

Cytotoxicity % = {(EV − LC/HC − LC)}100(5)

where EV = experimental value, LC = low control, and HC = high control.

### 4.8. Statistical Analysis

All results are presented as mean ± standard deviation (SD), and the studies were conducted in triplicate. One-way ANOVA was used to analyze the data using SPSS software (version 21.0).

## 5. Conclusions

EOs have also recently shown success in the treatment and prevention of cancer. This study investigated the comparative anticancer activity and cytotoxic effect of the plant extract and EOs for the first time. The EOs of the endemic species *T. fedtschenkoi* var. *handelii* have been found to be a rich source of carvacrol and, together with gamma-terpinene and p-cymene, exhibit strong anticancer properties. Consequently, *T. fedtschenkoi* var. *handelii* is a promising in vitro candidate for the development of novel and effective phytotherapy agents, particularly against aggressive strains such as prostate and bone cancer. Future in vivo studies hold critical data for clarifying the clinical potential of this endemic species.

## Figures and Tables

**Table 1:** EO content of *Thymus fedtschenkoi* var. *handelii*.

No.	RT (Minutes)	Compounds	Percent %	RI
**1**	3.972	Isovalerate <methyl->	0.03	773
**2**	8.459	(+)-4-Carene	0.01	919
**3**	8.722	Thujene <alpha->	1.40	927
**4**	8.934	alpha-Pinene	1.06	930
**5**	9.472	Camphene	0.31	943
**6**	10.576	Sabinene	0.28	972
**7**	11.308	Myrcene	2.06	991
**8**	11.734	alpha-Terpinene	0.32	998
**9**	11.949	Phellandrene <alpha->	0.11	1009
**10**	12.243	Carene <delta-3->	2.93	1012
**11**	12.629	Cymene <para->	14.76	1025
**12**	13.604	Ocimene <(E)-, beta->	0.04	1046
**13**	14.022	Terpinene <gamma->	16.87	1058
**14**	14.344	Sabinene hydrate <cis->	0.04	1069
**15**	15.096	Terpinolene	0.14	1086
**16**	15.702	Linalool	0.29	1087
**17**	16.489	Menth-2-en-1-ol-trans-para	0.04	1109
**18**	18.216	Isoborneol	0.87	1162
**19**	18.641	trans-beta-Terpineol	0.86	1163
**20**	19.381	Dihydrocarveol	0.19	1196
**21**	23.135	p-Cymen-7-ol	0.09	1284
**22**	23.348	Thymol	2.62	1300
**23**	23.863	Carvacrol	51.12	1317
**24**	27.271	Elemene <beta->	2.59	1398
**25**	27.903	Caryophyllene <beta->	0.06	1418
**26**	28.388	Bergamotene <alpha-trans->	0.09	1432
**27**	28.620	Alloaromadendrene	0.00	1458
**28**	29.740	Viridiflorene	0.04	1491
**29**	30.354	Cadinene <gamma->	0.01	1512
**30**	30.630	Cadinene <delta->	0.01	1518
**31**	32.413	Spathulenol	0.11	1576
**32**	32.525	Cubenol	0.55	1580
**33**	33.330	Caryophyllene oxide	0.01	1587
**34**	34.296	Eudesmol <gamma->	0.05	1632
**35**	35.246	beta-Santalol	0.03	1654
**36**	39.867	E-8-Methyl-9-tetradecen-1-ol acetate	0.01	1822

RI: retention indices calculated against n-alkanes; RT: retention time, % calculated from FID data.

**Table 2:** GI_50_, TGI, and LC_50_ values of test molecules on cancer and normal cells *, **, ***.

**(µg/mL)**	**A549**			**Calu1**			**H1650**			TSI
	GI_50_	TGI	LC_50_	GI_50_	TGI	LC_50_	GI_50_	TGI	LC_50_	
**Extract**	1.43	255.9 ^± 17^	>500	2.09	192.6 ^± 7^	>500	2.34	173.1 ^± 9^	>500	0.97
**EOs**	2.03	23.08 ^± 2^	>500	1.20	6.55	>500	1.13	2.87	>500	0.44
**5FU**	1.27	58.17 ^± 4^	403.2 ^± 21^	1.44	65.74 ^± 4^	455.8 ^± 19^	1.66	53.97 ^± 4^	417.2 ^± 21^	1.11
	**SW1353**			**MG63**			**Saos2**			
	GI_50_	TGI	LC_50_	GI_50_	TGI	LC_50_	GI_50_	TGI	LC_50_	
**Extract**	1.80	148.7 ^± 8^	>500	2.27	110.6 ^± 6^	>500	1.93	182.8 ^± 9^	>500	1.36
**EOs**	1.38	9.19	>500	1.62	8.42	>500	1.05	1.97	>500	0.74
**5FU**	1.38	48.8 ^± 3^	488.7 ^± 18^	1.63	56.78 ^± 2^	389.0 ^± 16^	1.61	49.66 ^± 2^	377.6 ^± 15^	1.27
	**A172**			**B35**			**C6**			
	GI_50_	TGI	LC_50_	GI_50_	TGI	LC_50_	GI_50_	TGI	LC_50_	
**Extract**	1.73	141.0 ^± 9^	>500	3.28	202.6 ^± 14^	>500	2.51	167.2 ^± 7^	>500	1.18
**EOs**	1.45	6.51	>500	1.23	3.75	>500	1.27	5.63	>500	0.91
**5FU**	1.41	68.29 ^± 5^	369.5 ^± 18^	1.57	72.79 ^± 4^	451.3 ^± 23^	1.45	68.50 ^± 3^	418.8 ^± 21^	0.94
	**PC3**			**DU145**			**LNCaP**			
	GI_50_	TGI	LC_50_	GI_50_	TGI	LC_50_	GI_50_	TGI	LC_50_	
**Extract**	1.79	147.4 ^± 8^	>500	2.09	166.1 ^± 7^	>500	1.10	132.2 ^± 9^	>500	1.35
**EOs**	1.24	3.56	>500	1.61	5.55	>500	1.19	4.09	>500	1.10
**5FU**	1.17	78.38 ^± 4^	344.2 ^± 16^	1.23	49.52 ^± 2^	401.0 ^± 23^	1.25	55.93 ^± 3^	420.1 ^± 24^	1.07
	**Beas2B**			**FL**			**HC**			
	GI_50_	TGI	LC_50_	GI_50_	TGI	LC_50_	GI_50_	TGI	LC_50_	
**Extract**	2.78	130.7 ^± 6^	>500	3.01	211.3 ^± 11^	>500	2.45	261.5 ^± 9^	>500	
**EOs**	1.16	2.62	>500	1.71	7.46	>500	1.39	4.49	>500	
**5FU**	1.33	52.75 ^± 3^	388.2 ^± 14^	1.51	58.94 ^± 2^	354.7 ^± 15^	1.53	85.90 ^± 4^	329.1 ^± 16^	

* Percent inhibition noted is mean values ± SDs of three independent measures. ** If percent inhibition is smaller than 10, the SD value is <0.5. *** Differences were considered statistically significant at *p* < 0.05.

**Table 3:** IC_50_ values of test molecules on cancer and normal cells *, **, ***.

(µg/mL)	A549	Calu1	H1650	SW1353	MG63	Saos2	A172	B35	C6
**Extract**	64.8 ^± 3^	81.2 ^± 4^	76.3 ^± 3^	82.8 ^± 4^	72.8 ^± 3^	77.5 ^± 4^	63.8 ^± 4^	72.4 ^± 3^	72.8 ^± 3^
**EOs**	51.4 ^± 2^	38.6 ^± 1^	16.4 ^± 1^	40.6 ^± 2^	41.4 ^± 2^	3.1	35.8 ^± 1^	24.6 ^± 1^	35.6 ^± 2^
**5FU**	83.1 ^± 3^	72.4 ^± 4^	79.8 ^± 2^	63.2 ^± 3^	70.3 ^± 4^	81.6 ^± 5^	77.5 ^± 3^	73.9 ^± 4^	71.5 ^± 4^
	**PC3**	**DU145**	**LNCaP**	**Beas2B**	**FL**	**HC**			
**Extract**	70.3 ^± 3^	71.4 ^± 4^	80.5 ^± 4^	66.9 ^± 3^	72.7 ^± 3^	78.2 ^± 4^			
**EOs**	23.6 ^± 1^	35.9 ^± 1^	27.8 ^± 1^	7.9	39.8 ^± 1^	30.4 ^± 2^			
**5FU**	59.4 ^± 2^	62.0 ^± 3^	57.3 ^± 2^	68.0 ^± 3^	65.1 ^± 3^	57.8 ^± 2^			

* Percent inhibition noted is mean values ± SDs of three independent measures. ** If percent inhibition is smaller than 10, the SD value is <0.5. *** Differences were considered statistically significant at *p* < 0.05.

**Table 4:** Percent cytotoxicity values of samples against cells at TGI concentrations *, **, ***.

% Cytotoxicity	A549	Calu1	H1650	SW1353	MG63	Saos2	A172	B35	C6
**Extract**	7.2	5.3	1.2	2.9	3.8	9.7	7.6	4.5	1.8
**EOs**	13.9 ^± 1.7^	17.3 ^± 1.8^	15.1 ^± 2.1^	18.0 ^± 2.2^	14.1 ^± 1.7^	18.8 ^± 2.2^	17.1 ^± 1.9^	14.9 ^± 1.5^	16.4 ^± 2.1^
**5FU**	8.3	11.2 ^± 1.5^	14.6 ^± 1.7^	12.3 ^± 1.3^	13.0 ^± 1.5^	14.5 ^± 1.9^	13.6 ^± 1.3^	12.7 ^± 1.5^	10.5 ^± 1.1^
	**PC3**	**DU145**	**LNCaP**	**Beas2B**	**FL**	**HC**			
**Extract**	4.0	3.5	9.8	4.1	8.4	8.8			
**EOs**	17.5 ^± 2.0^	18.2 ^± 1.7^	18.5 ^± 2.0^	17.3 ^± 2.1^	15.8 ^± 1.4^	16.9 ^± 1.9^			
**5FU**	14.6 ^± 1.9^	14.1 ^± 1.2^	9.5	10.9 ^± 1.1^	13.7 ^± 1.4^	10.0 ^± 1.3^			

* Percent cytotoxicity was noted as mean values ± SDs of three independent measures. ** If percent cytotoxicity is smaller than 10, the SD value is <0.5. *** Differences were considered statistically significant at *p* < 0.05.

## Data Availability

The original contributions presented in this study are included in this article; further inquiries can be directed to the corresponding author.

## Supplementary Materials

The following supporting information can be downloaded at: https://www.mdpi.com/article/10.3390/ph19060844/s1, Figure S1: MTT results of the *Thymus fedtschenkoi* var. *handelii*.
