# Supplementary material for: Phytochemical Characterization and the First Report on the Antiproliferative Activity and Cytotoxicity of Thymus fedtschenkoi var. handelii (Ronniger) Jalas
Source: Pharmaceuticals (Basel). 2026 May 28;19(6):844. doi: 10.3390/ph19060844 (PMC13306204; doi:10.3390/ph19060844)
Supplement: Supplementary file 1 [file pharmaceuticals-19-00844-s001.zip › pharmaceuticals-4303823-supplementary.pdf]

Supplemental Data

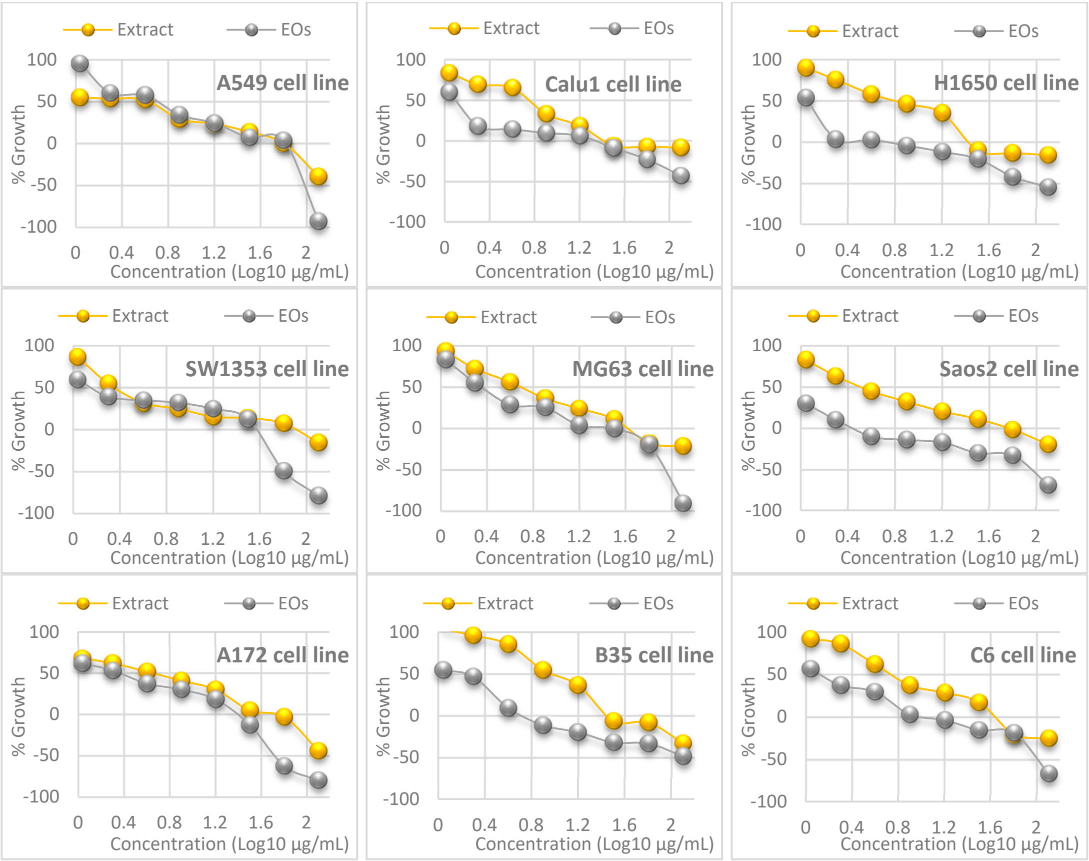

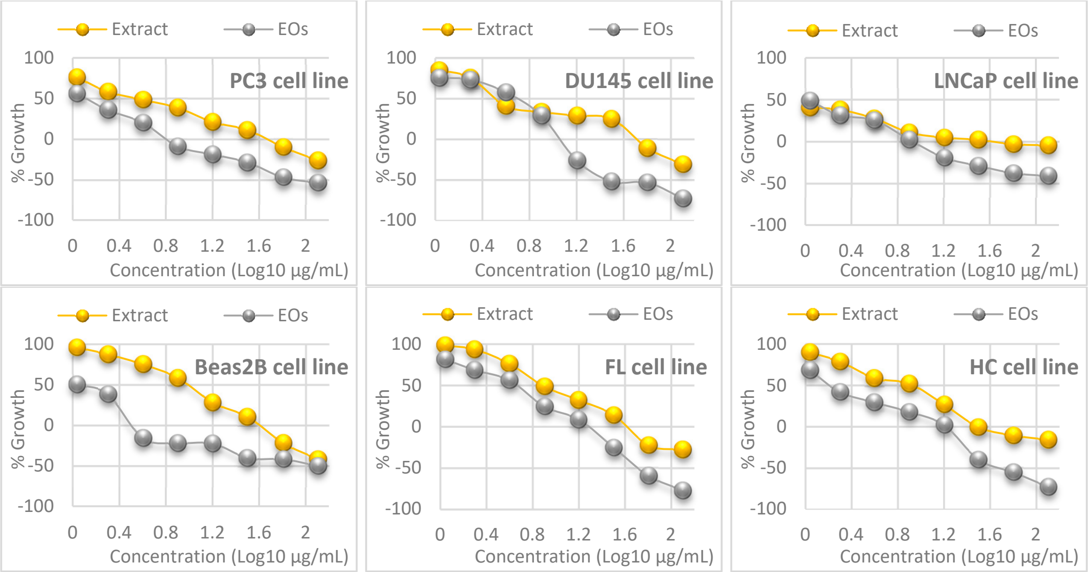

Supplementary Figure S1. MTT results of the *Thymus fedtschenkoi* var. *handelii.*
